# AMSA-Net: attention-based multi-scale feature aggregation network for single image dehazing

**DOI:** 10.3389/fnbot.2026.1698100

**Published:** 2026-02-17

**Authors:** Shanqin Wang, Mengjun Miao, Miao Zhang

**Affiliations:** 1 School of Information Engineering, Chuzhou Polytechnic, Chuzhou, China; 2 School of Computer, Qinghai Normal University, Xining, China

**Keywords:** haze density, hybrid attention, multi-scale feature refinement, scale-aware, single image dehazing, spatial feature

## Abstract

**Problem:**

Deep learning technology promotes the development of single-image dehazing. However, many existing methods fail to fully consider the haze density and its spatial distribution, which limits the improvement of dehazing performance.

**Proposed solution:**

To address this issue, we propose an attention-based multi-scale feature aggregation network (AMSA-Net) for single-image dehazing.

**Method:**

AMSA-Net is an encoding and decoding structure. Its encoder and decoder are composed of multi-scale hybrid attention feature aggregation module (MSHA-FAM). The module can perceive the haze density and spatial information in the haze image, which helps to improve the dehazing effect. MSHA-FAM is composed of two key components: the scale-aware coordinate residual module (SCRM) and multi-scale feature refinement residual module (MSFRRM). SCRM uses improved coordinate attention to effectively capture haze density and spatial characteristics, thus significantly improving dehazing effect. MSFRRM extracts semantic features through up-sampling and down-sampling, and uses improved pixel attention mechanism to enhance key features. In the overall MSHA-FAM pipeline, SCRM first learns the density and spatial distribution characteristics of haze, then refines it through MSFRRM, so as to remove haze more effectively.

**Key results:**

The experimental results demonstrate that our proposed AMSA-Net is superior to the comparison methods in terms of dehazing quality. Ablation studies further verify the effectiveness of the proposed modules.

**Impact:**

In this work, we present AMSA-Net, which has achieved good dehazing performance and can provide high-quality input for subsequent computer vision tasks.

## 1 Introduction

Outdoor images are easily affected by weather conditions in the shooting process. In hazy environments, atmospheric scattered particles can reduce visibility, blur scene details and mask real image features. This degradation typically results in low contrast, color distortion, and blurring, which pose significant challenges to the performance of advanced computer vision applications, such as intelligent driving (Zhang and Sun, 2024), video surveillance (Duong et al., 2023), and object detection (Sun et al., 2024). Therefore, single image dehazing technology has received extensive attention. The goal of this task is to restore the haze image to a clear image.

With the development of image-dehazing technology, many dehazing methods have been developed. Early methods are mainly based on the atmospheric scattering models (Narasimhan and Nayar, 2003) for dehazing. Specifically, these methods estimate atmospheric light and transmission values by statistical prior knowledge in the image to restore a clear image (He et al., 2010; Bui and Kim, 2017). Although these methods have achieved some success in dehazing, they do not dehazing according to the degradation factors of the image, resulting in the dehazing effect to be improved. In addition, when the statistical prior knowledge is not accurate, it will lead to poor dehazing effect.

The emergence of deep learning technology has prompted researchers to develop data-driven methods. Many technologies have become basic components in the design of dehazing models, such as the attention mechanism that focuses on important areas of images (Guo M. H. et al., 2022; Ma et al., 2022; Dai et al., 2023), the residual structure that promotes the transfer of features from shallow to deep (Ji et al., 2023; Sheng et al., 2022), and the large kernel convolution structure that can learn the characteristics of large receptive fields of images (Li et al., 2024; Moon and Park, 2025). These components are combined in different ways to design dehazing networks with different characteristics, such as encoder decoder networks (Tran et al., 2022; Tran and Park, 2025; Yang et al., 2022) and other multi-scale feature aggregation networks (Wu et al., 2025; Zhang et al., 2025). These innovations have greatly promoted the development of image dehazing technology and achieved excellent dehazing effect. However, many existing methods cannot fully explore the haze density information and its spatial distribution in the image. This will lead to incomplete haze removal or residual artifacts in the restored image. These artifacts, such as unnatural color changes and halos around edges, reduce the visual quality and realism of the output, which will affect the accuracy of subsequent computer vision tasks. Therefore, it is still a challenge to design effective models to restore high-quality images.

In order to restore high-quality images, an attention-based multiscale feature aggregation network, namely AMSA-Net, is proposed for single-image dehazing. This network can effectively perceive the haze density characteristics of the degraded image, and also learn its spatial correlation characteristics. AMSA-Net uses codec structure for dehazing. Its encoder and decoder are composed of multi-scale hybrid attention feature aggregation module (MSHA-FAM), and uses a physics-aware intra level fusion module (PIFM) for hierarchical feature fusion. MSHA-FAM synergistically integrates the scale-aware coordinate residual module (SCRM) and multi-scale feature refinement residual module (MSFRRM) through residual connections, effectively modeling haze density distributions and their spatial dependencies while implementing adaptive feature refinement. Specifically, SCRM first uses different kernel convolutions to learn the haze density features of the image and then employs improved coordinate attention (CA) to learn the corresponding positional information. MSFRRM first uses up-sampling and down-sampling operations to obtain contextual information and then employs a residual feature extraction block (RFEB) for feature learning. Subsequently, it uses enhanced pixel-wise attention (EPA) to focus on the important features. The main contributions of this study are summarized as follows:

1. This paper proposes a novel attention-based multi-scale feature aggregation network for single-image dehazing, which combines attention mechanism and multiscale features to effectively capture haze concentration and spatial distribution.
2. This study proposes SCRM, which leverages multi-scale convolutions and CA to capture haze density distribution and spatial features, significantly enhancing the dehazing performance.
3. This study proposes MSFRRM, which utilizes residual blocks in conjunction with down-sampling and up-sampling operations to achieve semantic feature aggregation.

## 2 Related work

### 2.1 Traditional-based methods

Traditional-based dehazing methods have demonstrated limited yet non-negligible effectiveness in the field. He et al. (2010) introduced a pioneering framework based on a physical prior known as the dark channel prior (DCP). By integrating physical models with statistical priors, their study laid the foundation for the development of numerous advanced dehazing algorithms. Bui and Kim (2017) proposed a method in which chromatic ellipsoids were constructed in the RGB color space by statistically fitting hazy pixel clusters. Through the geometric analysis of these ellipsoids, the transmission ratio is obtained. This method produces a transmission map, which can maximize the contrast of the dehazing output and effectively prevent pixel oversaturation. Zhao (2021) observed systematically that when the estimated transmission value deviates from the ground truth value by 1, the output of the measurement function decreases accordingly. The metric is derived using the difference map derived from the three RGB channels of the local denoised image block normalized by the global atmospheric light. Therefore, a new single-image dehazing method is proposed. It divides the image into several blocks, accurately estimates the transmission value in each block, and applies weighted interpolation and guided filtering to enhance the edges and details of the rough transmission image, which makes it obtain better dehazing effect. Ling et al. (2023) put forward the saturation line prior (SLP) according to their observation that the saturation information in the blurred image plays an important role in the effective removal of haze. This is a new dehazing method, which can restore details and realistic color reproduction in hazy scenes, while significantly enhancing the overall visibility. Dwivedi and Chakraborty (2023) proposed an enhanced dark channel calculation method based on the selection of the minimum intensity per pixel on the RGB channel, which can alleviate the inherent non-uniform estimation limitation in the traditional DCP implementation. Although these early dehazing methods have achieved some success in removing haze, their generalization performance needs to be improved due to their strong dependence on statistical priors.

### 2.2 Learning-based methods

Deep learning technology has promoted the development of single image defogging task, especially in recent years, single image dehazing method based on learning has developed rapidly. In order to learn the complex mapping of fuzzy input into clear images, Xiao et al. (2024) combined with Laplacian pyramid, proposed LapDehazeNet. Liu et al. (2023) proposed a new dehazing method, which uses a dynamically activated dehazing function based on global context coding to eliminate the haze and haze superposition effect. In addition, in order to reduce the loss of detail, the method uses dynamic dehazing convolution operation with attention mechanism and dynamic weight fusion to design the model. Ju et al. (2024) used the multi-scale gated fusion module to design a fully guided dehazing network, which can adjust the information flow between the characteristic graphs at different decoding stages. The network employs CNN-transformer dual-branch blocks as its fundamental building units to achieve a detailed image reconstruction. Zhu et al. (2023) proposed a spectrum guided enhancement framework based on the frequency sensing dehazing principle. The framework uses spectrum energy coding to decompose the blurred image into high-frequency and low-frequency components, and uses a specially designed network architecture to restore the degradation characteristics in each frequency domain. Yin et al. (2024) designed two branch network based on ordinary differential equation (ODE). The network effectively combines visual attention module with ode inspired network and prior guidance, and achieves good haze removal effect through physical information feature fusion. Zheng et al. (2023) designed the dehazing model by contrastive learning strategy to reduce the recovery ambiguity in the training process and encourage the model to converge in the constrained solution space, so as to promote stable optimization. In order to enable the decoding layer to integrate shallow and deep features, Sun et al. (2023) designed a dehazing model with multi-level feature interaction and nonlocal enhanced channel attention by using the multi-level feature interaction module. Although these methods have achieved certain dehazing effect in single image task, the dehazing effect needs to be further improved because they fail to fully explore the density distribution characteristics and spatial location information of haze. Therefore, in order to further improve the dehazing effect, this paper proposes AMSA-Net, which can effectively perceive the distribution characteristics of haze density and spatial location information, so as to promote the network to achieve effective dehazing.

## 3 Method

### 3.1 Overall structure

**Figure 1:**
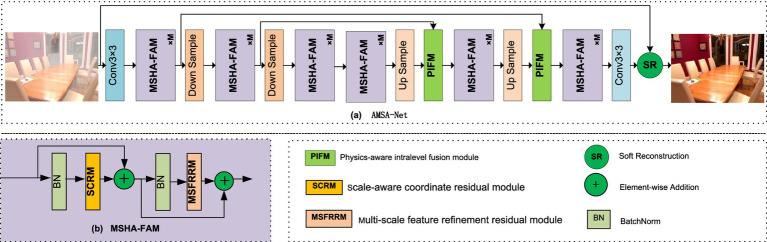
Overall framework of the proposed AMSA-Net.

Effectively perceiving the density of haze and its location information helps improve image dehazing. Therefore, we developed a novel network for single-image dehazing, AMSA-Net, which is an attention-based multi-scale feature aggregation network. As shown in Figure 1, the architecture of AMSA-Net comprises two main stages: encoding and decoding. The model first encodes the input through successive down-sampling layers and feature extraction to extract hierarchical features, then decodes them via a series of up-sampling layers to progressively reconstruct the output. In this process, the PIFM (Wen et al., 2023) is employed to fuse the features from different levels of the encoder with the corresponding decoder. By integrating a physical scattering model, PIFM effectively enhances the interpretability of the network. In addition, soft residual connections (Song et al., 2023) are employed to improve the model performance. Both the encoder and decoder are constructed using a stack of MSHA-FAM. It achieves good dehazing performance through the collaborative integration of multiscale feature fusion and a coordinate-guided attention mechanism. During the encoding phase, the number of channels doubles while the spatial dimensions (width and height) are halved at each layer. The decoding phase follows the opposite pattern.

### 3.2 MSHA-FAM

MSHA-FAM serves as a plug-and-play module for dehazing. It enhances dehazing performance through haze density perception and effective feature aggregation. As shown in Figure 1, MSHA-FAM consists of BatchNorm, residual connections, and the proposed SCRM and MSFRRM. Specifically, SCRM significantly improves dehazing performance by utilizing the CA mechanism. The MSFRRM utilizes RFEB, down-sampling, and up-sampling operations to achieve semantic feature aggregation. MSHA-FAM first obtains the feature map $x^{\prime }$ using BatchNorm, SCRM, and residual connection. Subsequently, $x_{\text{out}}$ is obtained through BatchNorm, the MSFRRM and the residual connection. The specific mathematical expression is shown in Equations 1, 2:


$$x^{\prime }=\text{SCRM}(\mathrm{BN}(x_{\text{in}}))+x_{\text{in}},$$ (1)

$$x_{\text{out}}=\text{MSFRRM}(\mathrm{BN}(x^{\prime }))+x^{\prime }.$$ (2)

where, $x_{\text{in}}$ , $x^{\prime }$ , and $x_{\text{out}}$ represent the input, intermediate, and output features, respectively. *BN* represents the BatchNorm.

### 3.3 SCRM

**Figure 2:**
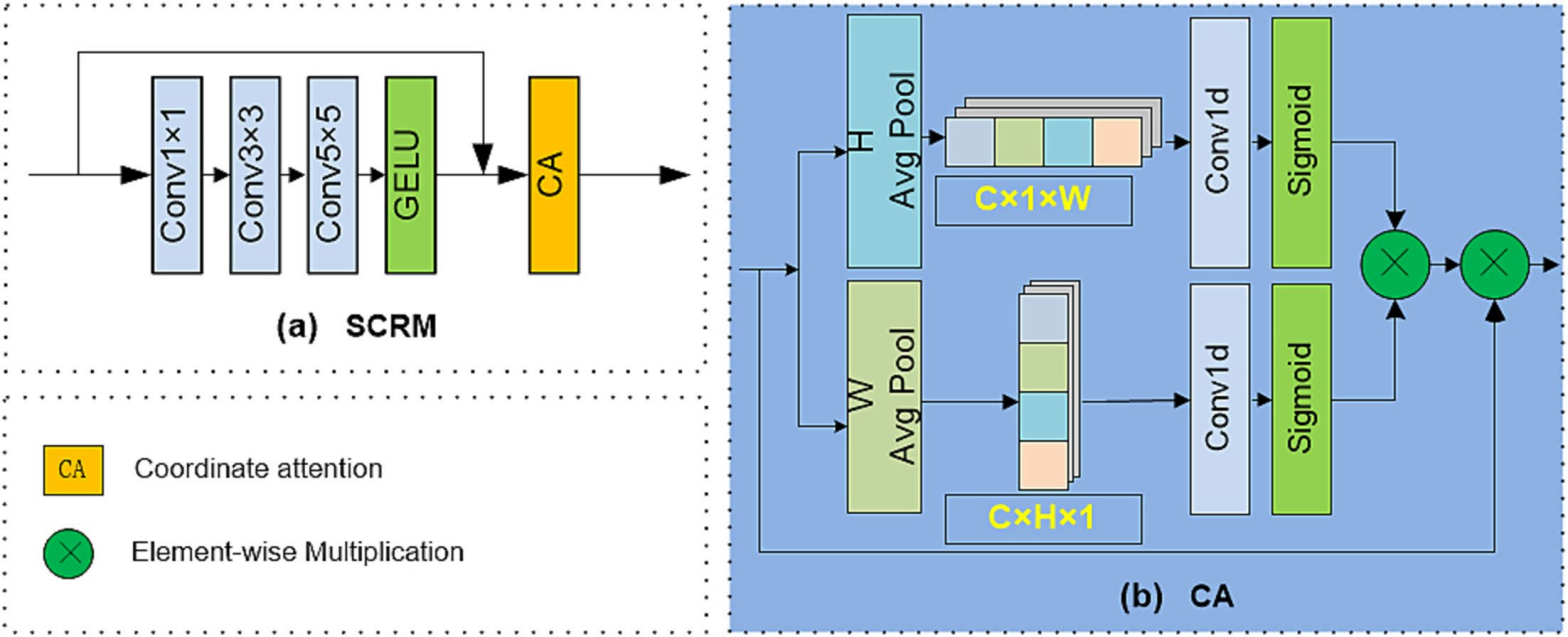
Structure diagram of SCRM.

SCRM utilizes a multi-branch convolution structure with different kernel sizes for comprehensive spatial feature extraction, and adopts a new CA mechanism to generate haze density characteristics. This design can promote the restoration of image detail features to improve dehazing performance. Unlike the CA described in reference (Huang et al., 2024), our method introduces separate 1D convolutional layers for each path before fusion, instead of directly connecting the merged horizontal and vertical features. This design allows for more refined and independent processing of spatial dependencies, thereby more effectively capturing long-distance cross channel information along each coordinate. As shown in Figure 2, it first employs convolutions with multiple different kernels to learn the haze density features of the image, and then employs CA to learn the corresponding positional information, thereby capturing the haze density distribution and spatial features and significantly improving the dehazing performance. Specifically, input *x* first underwent convolutions with 1×1, 3×3 , and5×5 kernels, followed by the GELU activation function, and a residual connection was applied to obtain the $f^{\prime }$ . Subsequently, we utilized CA to learn the positional features, obtaining $f_{\text{out}}$ . The specific mathematical expression is shown in Equations 3, 4:


$$f^{\prime }=\text{GELU}(\mathrm{Con}v_{5\times 5}(\mathrm{Con}v_{3\times 3}(\mathrm{Con}v_{1\times 1}(x))))+x,$$ (3)

$$f_{\text{out}}=\mathrm{CA}(f^{\prime }).$$ (4)

where $\mathrm{Con}v_{k\times k}$ indicates a convolution with a kernel of the size k×k , GELU is the GELU activation function, and CA is the proposed coordinate attention, as shown in Figure 2. First, average pooling operations is applied in the width and height directions, then a one-dimensional convolution and Sigmoid activation function are applied to each; that is, multiply the two results to obtain the weight w, and then with the input feature map $f^{\prime }$ to obtain the coordinate attention feature map *F*. The specific mathematical expression is shown in Equations 5, 6:


$$\begin{array}{l} w=\sigma (\text{Conv}1d(\text{AvgPoo}l_{H}(f^{\prime })))\cdot \\ (\sigma (\text{Conv}1d(\text{AvgPoo}l_{W}(f^{\prime })))), \end{array}$$ (5)

$$F=w\times f^{\prime }.$$ (6)

where σ denotes the Sigmoid activation function, $\text{AvgPoo}l_{H}$ and $\text{AvgPoo}l_{W}$ are average pooling operations on the height and width directions, respectively, and Conv1d is one-dimensional convolution operation.

### 3.4 MSFRRM

**Figure 3:**
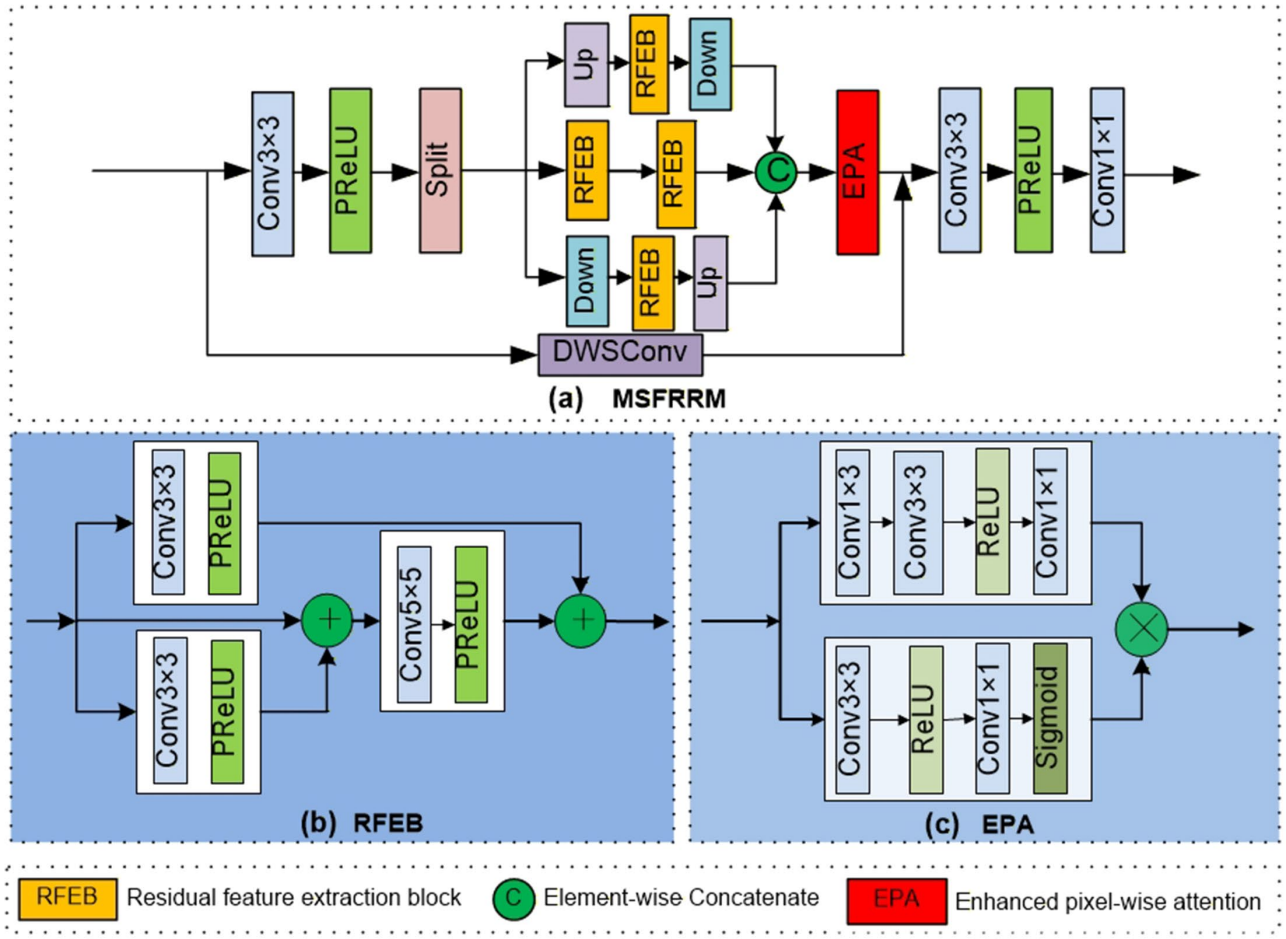
Structure diagram of MSFRRM.

The MSFRRM is designed with multiple parallel branches to extract multi-scale features directly within each layer. The key design is the application of up-sampling and down-sampling operations within the same layer, which allows for immediate capture of characteristics at various scales. It also employs a RFEB to learn robust semantic representations and an enhanced pixel-wise attention (EPA) module to refine the feature output. The proposed EPA module introduces a key difference from conventional pixel attention: by operating on the features produced by the feature extraction block rather than on the original input features, it more effectively enhances informative regions in a transformed feature space. As shown in Figure 3, the MSFRRM first performs a 3×3 convolution, followed by the application of the PReLU activation function to obtain $f_{r}$ . Subsequently, the output is divided into three parts in the channel dimension to obtain $f_{r}^{1}$ , $f_{r}^{2}$ , and $f_{r}^{3}$ : the first part $f_{r}^{1}$ is processed through up-sampling, RFEB, and down-sampling to obtain $f_{r}^{1^{\prime }}$ ; the second part $f_{r}^{2}$ is performed via two layers of RFEB without up-sampling or down-sampling to obtain $f_{r}^{2^{\prime }}$ ; and the third part $f_{r}^{3}$ is processed through down-sampling, RFEB, and up-sampling to obtain $f_{r}^{3^{\prime }}$ . The output results of the three paths were merged through concatenation, followed by EPA to obtain ${f_{r}}^{\prime }$ . Subsequently, the output result is merged with the result processed using depthwise separable convolution via a residual connection to obtain ${f_{r}}^{\prime \prime }$ . Finally, a 3×3 convolution, PReLU activation function, and 1×1 convolution are applied to further refine the features to obtain $f_{r}^{\text{out}}$ .

The specific mathematical expression is shown in Equations 7–14:


$$f_{r}=P(\mathrm{Con}v_{3\times 3}(x)),$$ (7)

$$f_{r}^{1},f_{r}^{2},f_{r}^{3}=\text{Split}(f_{r}),$$ (8)

$${f_{r}^{1}}^{\prime }=↓(R(↑(f_{r}^{1}))),$$ (9)

$${f_{r}^{2}}^{\prime }=R(R(f_{r}^{2})),$$ (10)

$${f_{r}^{3}}^{\prime }=↑(R(↓(f_{r}^{3}))),$$ (11)

$${f_{r}}^{\prime }=\Gamma (C({f_{r}^{1}}^{\prime }+{f_{r}^{2}}^{\prime }+{f_{r}^{3}}^{\prime }))$$ (12)

$$f_{r}^{\prime \prime }={f_{r}}^{\prime }+\text{DWSConv}(x),$$ (13)

$$f_{r}^{\text{out}}=\mathrm{Con}v_{1\times 1}(P(\mathrm{Con}v_{3\times 3}({f_{r}}^{\prime \prime }))).$$ (14)

where P is PReLU activation function, Split denotes the split operation, ↑ and ↓ represent up-sampling and down-sampling operations, respectively. *C* denotes concatenation operation, and *DWSConv* represents depthwise separable convolution. RFEB, as shown in Figure 3, consists of convolutions, a PReLU activation function, and residual connections. Γ refers to the proposed EPA module shown in Figure 3. Compared to the original pixel attention mechanism, EPA enhances features by applying multilayer convolutions and a rectified linear unit (ReLU) activation function to the original features; thus, effectively focusing on key features. The specific mathematical expression is shown in Equations 15–17:


$$w=\sigma ({\text{Conv}}_{1\times 1}(\text{ReLU}({\text{Conv}}_{3\times 3}(f_{x})))),$$ (15)

$${f_{x}}^{\prime }={\text{Conv}}_{1\times 1}(\text{ReLU}({\text{Conv}}_{3\times 3}({\text{Conv}}_{1\times 3}(f_{x})))),$$ (16)

$$\text{atten}=f_{x}^{\prime }\times w$$ (17)

where, *ReLU* is ReLU activation function, *σ* is Sigmoid activation function.

### 3.5 Loss function

The proposed AMSA-Net was optimized using *L*_1_ loss (*L*_1_) and structural similarity (SSIM) (Wang et al., 2003) loss (*L*_SSIM_). *L*_1_ focuses on the absolute differences in pixel values, whereas SSIM focuses on the similarities between images in terms of structure, brightness, and contrast. The combination of these two aids facilitated the model optimization.

The calculation of $L_{1}$ is shown in Equation 18:


$$L_{1}=\frac{1}{N}\sum_{i=1}^{N}|y_{i}-x_{i}|,$$ (18)

where *N* is the number of samples; $x_{i}$ and $y_{i}$ are the predicted value and ground truth, respectively.

The calculation of SSIM is shown in Equation 19:


$$\text{SSIM}(x,y)=\frac{\left(2\mu_{x}+C_{1})(2\sigma_{\mathrm{xy}}+C_{2}\right)}{\left(\mu_{x}^{2}+\mu_{y}^{2}+C_{1})(\sigma_{x}^{2}+\sigma_{y}^{2}+C_{2}\right)},$$ (19)

where, $\mu_{x}$ and $\mu_{y}$ are the mean pixel value of the recovered hazy-free image *x* and the corresponding ground truth *y*, respectively. $\sigma_{\mathrm{xy}}$ is the co-variance of *x* and y. $\sigma_{x}$ and $\sigma_{y}$ are the standard deviations of *x* and *y*, respectively.

We converted the SSIM value from maximum to minimum using Equation 20 to obtain *L_SSIM_*:


$$L_{\text{SSIM}}=1-\text{SSIM}(x,y),$$ (20)

This paper employs the combined loss of *L*_1_ and *L_SSIM_* for training. The specific formula is as shown in Equation 21:


$$L_{\text{total}}=L_{1}+L_{\text{SSIM}}$$ (21)

## 4 Experiment

### 4.1 Datasets and evaluation metrics

In our implementation, the proposed method is trained on both the indoor training set (ITS) and the outdoor training set (OTS) of the widely used RESIDE (Li et al., 2018) dataset, and evaluated on its synthetic objective testing set (SOTS). ITS contains 1,399 clear images and 13,990 hazy images generated by the corresponding clear images. OTS contains 8,970 clear images and 313,950 hazy images generated from the corresponding clear images. SOTS contains 500 image pairs of indoor and outdoor scenes.

The peak signal-to-noise ratio (PSNR) and structural similarity index (SSIM) (Setiadi, 2021) were employed to evaluate the performance of the proposed AMSA-Net and the comparative methods.

### 4.2 Experimental setup

Our model was trained on different datasets using an NVIDIA RTX 4090 GPU. For the ITS and OTS datasets, the image patch size used for training was 256 × 256 with a batch size of four, and the initial learning rate was set to 1 × 10^−4^, with updates performed via a cosine annealing learning rate scheduler. Training was conducted for 35 epochs in the OTS dataset and 350 epochs in the ITS dataset. Moreover, training was conducted with the AdamW optimizer, which is configured with default parameters ( $\beta_{1}$ and $\beta_{2}$ assume the default values of 0.9 and 0.999). In the inference phase, an NVIDIA GeForce RTX 4090 GPU is used with a batch size set to 1.

### 4.3 Comparative experiment

To evaluate the performance of the proposed method, we conducted a systematic comparison with 12 existing methods: DCP (He et al., 2010), AOD-Net (Li et al., 2017), GridDehazeNet (Liu et al., 2019), FFA-Net (Qin et al., 2020), Y-Net (Yang et al., 2020), MSBDN (Dong et al., 2020), AECR-Net (Wu et al., 2021), DeHamer (Guo C. L. et al., 2022), ODCR (Wang et al., 2024), CL2S (Rohn, 2024), Wang et al. (2025) and TransER (Hoang et al., 2023).

#### 4.3.1 Quantitative analysis

We compared the performance of AMSA-Net with those of the comparative methods, and the results are presented in Table 1. On the SOTS-Indoor dataset, AMSA-Net achieved the optimal performance in both PSNR and SSIM, reaching 38.05 dB and 0.9931 respectively, while TransER obtained the sub-optimal values. Compared with the sub-optimal values, the proposed method achieved a 0.81 dB increase in PSNR. On the SOTS-outdoor dataset, evaluation results show that the proposed method achieves the best SSIM, and although its PSNR is slightly lower than that of DeHamer, our model uses significantly fewer parameters. In summary, the proposed method achieves an excellent balance between dehazing performance and parameter efficiency. It provides the best performance on SOTS-indoor dataset with fewer parameters, while PSNR only shows an edge gap compared with the best performance method on the SOTS-outdoor dataset. This demonstrates that the method maintains high model efficiency without compromising its core performance. Furthermore, the parameter counts are calculated based on images of size 3 × 256 × 256. Although the parameter count of our method is not the lowest, it remains competitive. In future work, we will place further emphasis on the design of lightweight models.

**Table 1:** Quantitative analysis results on both SOTS-indoor and SOTS-outdoor datasets.

Method	SOTS-indoor		SOTS-outdoor		#Param (*M*)
	PSNR	SSIM	PSNR	SSIM	
DCP (He et al., 2010)	16.61	0.8546	19.14	0.8605	—
AOD-Net (Li et al., 2017)	19.06	0.8500	20.29	0.8765	**0.002**
GridDehazeNet (Liu et al., 2019)	32.16	0.9836	30.86	0.9819	0.956
FFA-Net (Qin et al., 2020)	36.39	0.9886	33.57	0.9840	4.456
Y-NET (Yang et al., 2020)	—	—	26.61	0.9470	0.197
MSBDN (Dong et al., 2020)	32.77	0.9812	34.81	0.9857	31.35
AECR-Net (Wu et al., 2021)	37.17	0.9901	—	—	2.61
DeHamer (Guo C. L. et al., 2022)	36.63	0.9881	**35.18**	0.9860	132.40
ODCR (Wang et al., 2024)	26.32	0.9450	26.16	0.9600	11.38
CL2S (Rohn, 2024)	35.36	0.9808	—	—	92.14
Wang et al. (2025)	28.40	0.9637	—	—	—
TransER (Hoang et al., 2023)	37.24	0.992			2.60
AMSA-Net	**38.05**	**0.9931**	34.92	**0.9889**	17.46

Bold indicates optimal value, underline indicates suboptimal value.

#### 4.3.2 Visualization analysis

**Figure 4:**
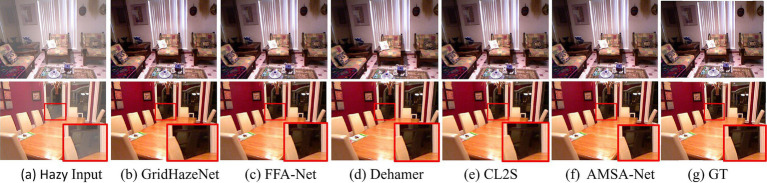
Visualization results on the SOTS-indoor dataset. Column **(a)** displays the hazy images; columns **(b–f)** display the dehazing results of different methods; column **(g)** displays the ground-truth (clear) images.

**Figure 5:**
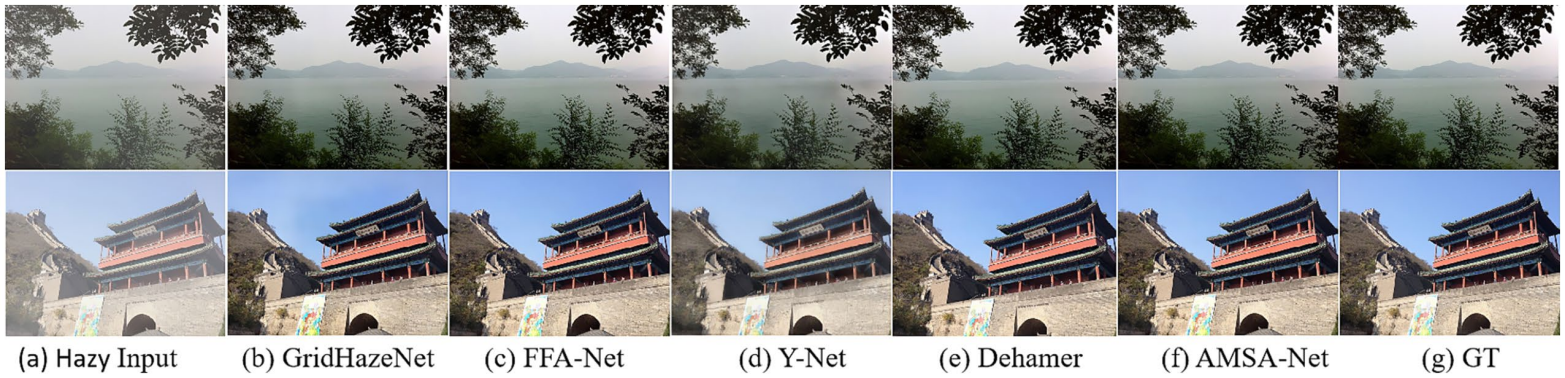
Visualization results on the SOTS-outdoor dataset. Column **(a)** displays the hazy images; columns **(b–f)** display the dehazing results of different methods; column **(g)** displays the ground-truth (clear) images.

Figures 4, 5 show the visual results of AMSA-Net and the partial comparison method for the SOTS-indoor and SOTS-outdoor datasets, respectively. As shown in Figure 4, the images restored by FFA-Net and Dehamer exhibited issues of color deviation and blurriness. In comparison with the ground truth, GridDehazeNet and CL2S introduced noticeable discrepancies in detail, particularly in the red area on the inner side of the chair back within the zoomed-in region. In contrast, the images restored by AMSA-Net were the clearest and closest to the ground truth. Similarly, as shown in Figure 5, Y-Net had haze residue on the SOTS-outdoor dataset, and the distribution of the residual haze in the restored image was uneven. GridHazeNet had artifacts in the sky region, whereas FFA-Net and Dehamer exhibit color differences in the sky region. In comparison, the restored images of AMSA-Net are clearer with less haze residue and sharper edge contours, and considerably close to the ground truth.

### 4.4 Ablation study

#### 4.4.1 Effectiveness of core components in AMSA-Net

We conducted a comprehensive ablation study to verify the effectiveness of the key modules in AMSA-Net. As presented in Table 2, w/o PIFM represents AMSA-Net with the PIFM removed, w/o SCRM refers to AMSA-Net from which the SCRM has been removed, and w/o MSFRRM denotes AMSA-Net after removing MSFRRM. The ablation study demonstrated the critical contributions of PIFM, SCRM, and MSFRRM to AMSA-Net. Specifically, removing the PIFM degrades the PSNR to 37.13 and the SSIM to 0.9918, whereas excluding the SCRM reduces the PSNR to 36.30 and the SSIM to 0.9922. After removing the MSFRRM, the performance of the model has the most significant decline, PSNR and SSIM are 32.86 and 0.9844, respectively. This shows that it plays an important role in multi-scale feature refinement. This is because the MSFRRM integrates up-sampling and down-sampling operations with RFEB to achieve effective multi-scale feature extraction. Furthermore, by incorporating EPA, it enhances the ability to capture critical features. The full model (AMSA-Net) achieved the best results, reaching 38.05 and 0.9931 on PSNR and SSIM respectively, which confirmed the necessity of all components for the best recovery quality.

**Table 2:** Ablation results on the effectiveness of core components in AMSA-Net on the SOTS-indoor dataset.

Model	SOTS-indoor	
	PSNR	SSIM
w/o PIFM	37.13	0.9918
w/o SCRM	36.30	0.9922
w/o MSFRRM	32.86	0.9844
AMSA-Net	38.05	0.9931

**Figure 6:**
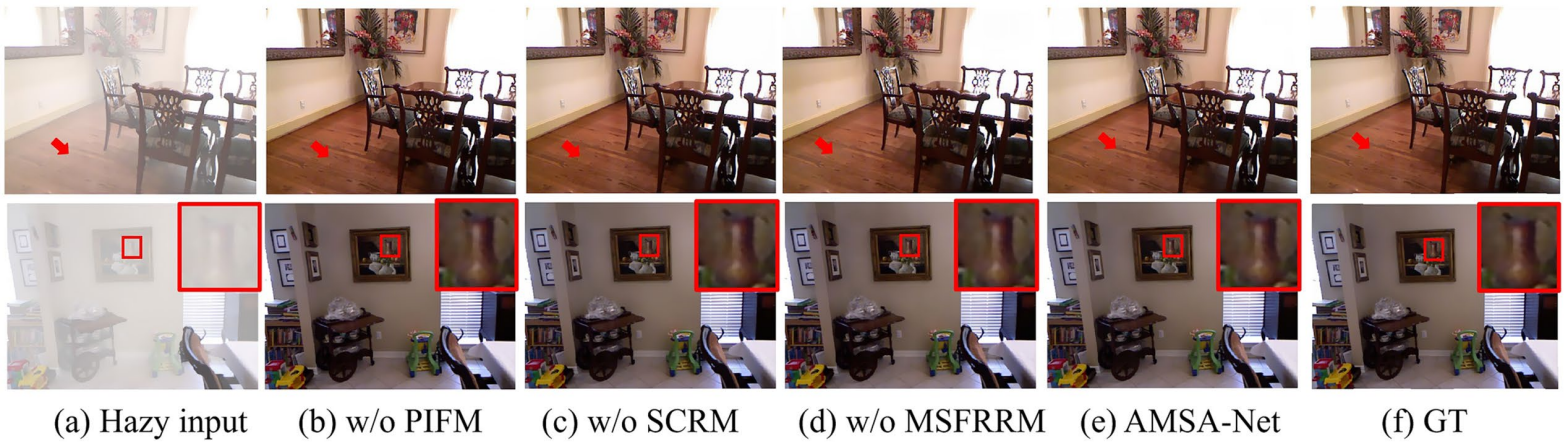
Visual comparison of the effectiveness of key components. Column **(a)** displays the hazy images; columns **(b–f)** display the dehazing results of different methods; column **(g)** displays the ground-truth (clear) images.

Figure 6 presents the visualization results of the core components. It can be observed that the lack of MSFRRM lead to obvious color deviation in the floor area indicated by the arrow in Sample 1, which is also proved in the wall area of the same sample. Compared to the ground truth, the results without SCRM and without PIFM exhibit visible artifacts and blurred details. In contrast, the image restored by the AMSA-Net is closer to the ground truth, which proves the effectiveness of these core components.

#### 4.4.2 Ablation study on the number *M* of encoders and decoders

In this section, we conduct ablation experiments on the number *M* of encoders and decoders. As shown in Table 3, when *M* is set to 1, the values of PSNR and SSIM reach 33.77 and 0.9888, respectively. When *M* is set to 2, the values of PSNR and SSIM reach 36.94 and 0.9924 respectively, demonstrating improvements in both metrics. At *M* is set to 3, the model achieved the best performance, with a PSNR of 38.05 and SSIM of 0.9931. Therefore, in this study, *M* is set to 3. Moreover, although increasing the number of encoder and decoder layers leads to a growth in model parameters, the substantial improvements in PSNR and SSIM metrics bring a qualitative leap in image dehazing performance, making this trade-off undoubtedly justified.

**Table 3:** Ablation results of the number *M* of encoders and decoders.

*M*	SOTS-indoor		#Param (*M*)
	PSNR	SSIM	
1	33.77	0.9888	5.87
2	36.94	0.9924	11.66
3	38.05	0.9931	17.46

**Figure 7:**
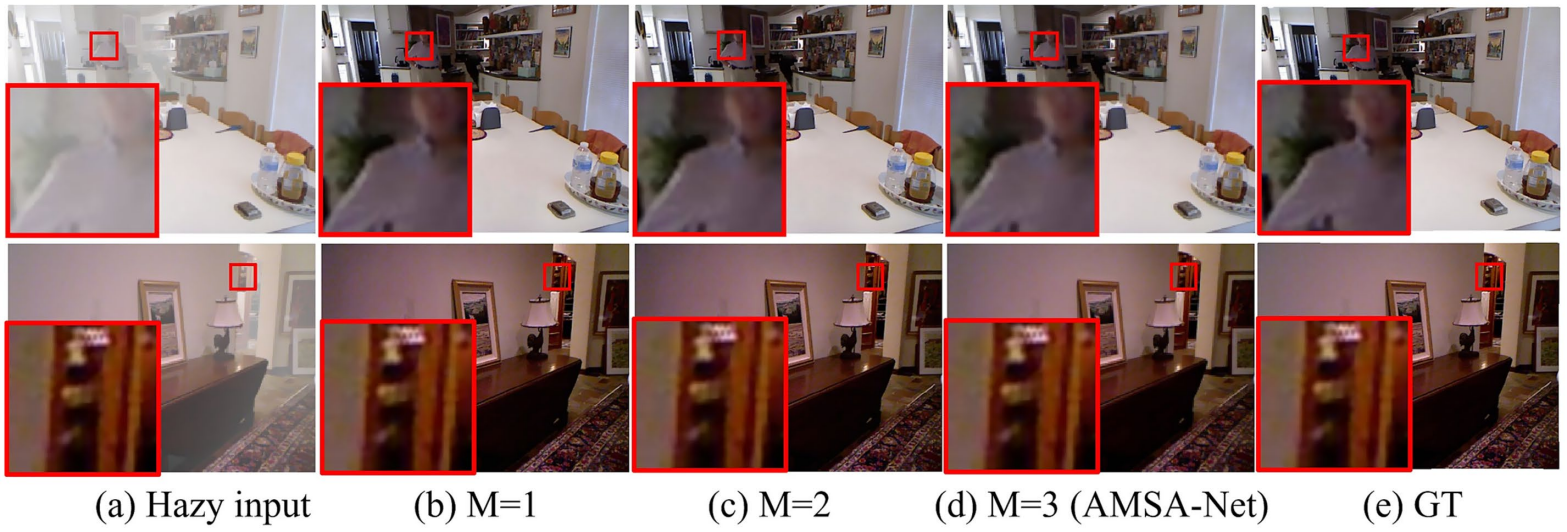
Visualization results for different *M* values. Column **(a)** displays the hazy images; columns **(b–d)** the dehazing results with different values of *M*; column **(e)** displays the ground-truth (clear) images.

Visual comparisons confirmed that higher *M* values significantly enhanced the image clarity and detail. As shown in Figure 7, in Sample 1, the neck area of the character in the enlarged region reveals distinct performance differences across methods: *M* = 1 and *M* = 2 struggle with haze removal, leaving faint artifacts; *M* = 3 achieved near-perfect dehazing with sharp edges and natural colors.

#### 4.4.3 Effectiveness of different loss functions

To verify the advantages of the joint loss used in this study, we used the *L*_1_, *L*_SSIM_, and *L*_1_ + *L*_SSIM_ joint losses, respectively, to observe the effect, with the specific results shown in Table 4. It can be seen that the *L*_1_ + *L*_SSIM_ loss achieved the best results, indicating that this method has advantages in taking into account the reconstruction accuracy and structural similarity. *L*_1_ achieved 35.98 and 0.9888 on PSNR and SSIM, respectively, slightly lower than *L*_1_ + *L*_SSIM_, indicating that introducing *L*_SSIM_ can further improve image quality. *L*_SSIM_ has poor performance with a PSNR of 28.19 and an SSIM of 0.9709, highlighting the importance of both pixel-wise and structural fidelity in image restoration. Therefore, this study combined *L*_1_ and *L*_SSIM_ to train the model.

**Table 4:** Ablation results on the effectiveness of different loss functions.

Model	SOTS-indoor	
	PSNR	SSIM
*L*_1_	35.98	0.9888
*L*_SSIM_	28.19	0.9709
*L*_1_ + *L*_SSIM_	38.05	0.9931

**Figure 8:**
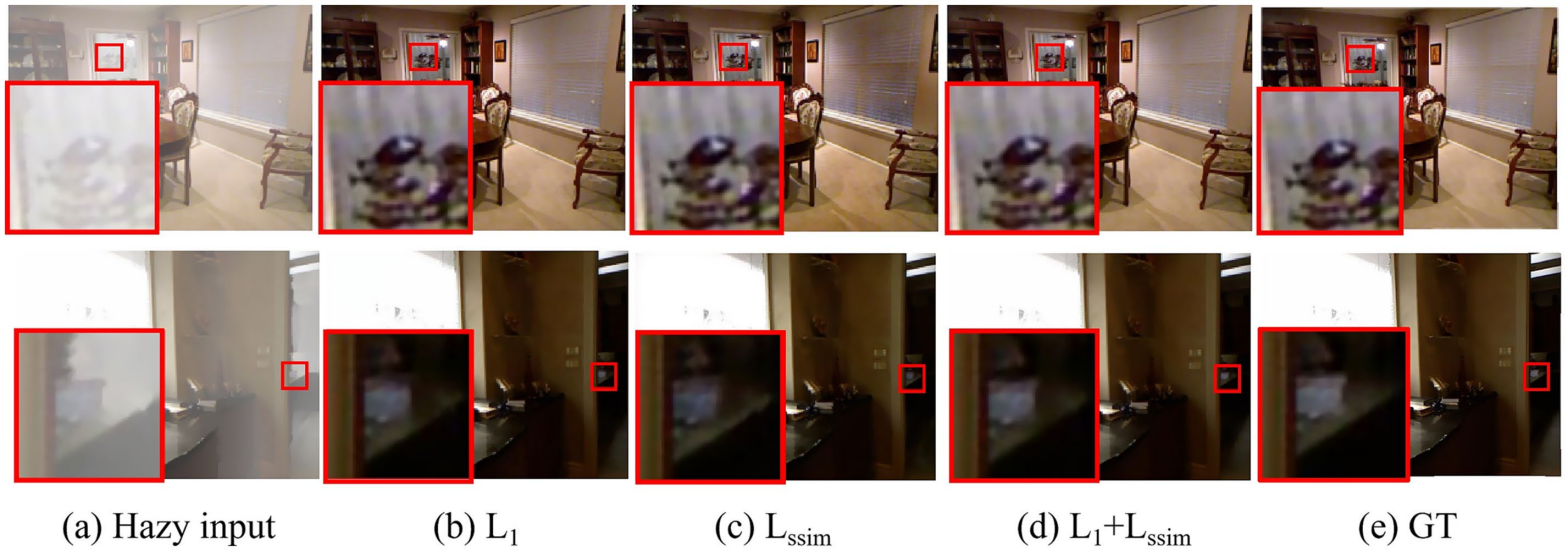
Visualization results of different loss functions. Column **(a)** displays the hazy images; columns **(b–d)** the dehazing results with different loss function; column **(e)** displays the ground-truth (clear) images.

As shown in Figure 8, in the white area of the enlarged region from Sample 2, *L*_1_ + *L*_SSIM_ loss produces images with clearer details, less noise, and better contrast, making it the preferred choice for dehazing tasks. In comparison, *L*_1_ exhibited certain noise, whereas the *L*_SSIM_ results in blurrier images with color shifts, which is consistent with quantitative research results.

## 5 Conclusion

AMSA-Net, an attention-based multi-scale feature aggregation network, is proposed for single-image dehazing. This network can perceive haze density, capture spatial information, and refine features, which makes it have a good dehazing effect. The proposed AMSA-Net uses encoding and decoding as the main architecture, MSHA-FAM for encoding and decoding, and PIFM for feature fusion. MSHA-FAM mainly consists of SCRM and MSFRM. SCRM first uses multi kernel convolution to extract haze density features, and then uses the proposed CA to capture corresponding positional information. The MSFRRM leverages up-sampling and down-sampling operations to acquire multi-scale contextual information, utilizes residual blocks for robust feature learning, and subsequently applies EPA to prioritize the salient features. Compared to existing methods, the proposed dehazing approach demonstrated superior performance on two public benchmark datasets. Furthermore, the ablation studies validated the efficacy of the core components of the network.

## Data Availability

The original contributions presented in the study are included in the article/supplementary material, further inquiries can be directed to the corresponding author.
